# P034. Technostress and primary headache: psychosocial risk

**DOI:** 10.1186/1129-2377-16-S1-A147

**Published:** 2015-09-28

**Authors:** Ennio Pucci, Silvano Cristina, Fabio Antonaci, Alfredo Costa, Marcello Imbriani, Giuseppe Taino

**Affiliations:** Headache Science Center, University Consortium for the Study of Adaptive Disorders and Headache (UCADH), Department of Brain and Behavioral Sciences, University of Pavia, IRCCS “C. Mondino”, Pavia, Italy; Department of Public Health, Experimental and Forensic Medicine, University of Pavia, Pavia, Italy; U.O. Hospital Occupational Medicine, Foundation IRCCS “S. Maugeri”, Pavia, Italy

## Introduction

Work and the working environment could have a decisive role in the development of symptoms which, in turn, may determine the onset of some forms of headache, as well as increase the frequency and/or intensity of pre-existing forms. Technostress is one of the new occupational diseases under the ruling of Judge Guariniello (2007) and has been included in the obligations of risk assessment in accordance with T.U. 81/2008 and Legislative Decree 106/2009. Under this term various addictions are covered: video addiction, internet addiction disorder, social network craze, information overload, multitasking, cybersex addiction, and email addiction. The psychologist Craig Brod was the first to coin this term, which can manifest itself with many symptoms: headache, hypertension, anxiety, panic attacks, loss of concentration, gastrointestinal and cardiovascular disorders, depression, loss of libido and even behavioral changes and relational isolation. In the past, this condition mostly affected the manager, it is now widespread among workers of other at-risk groups, such as, call center operators, accountants, networkers, journalists, advertisers and financial analysts. Training for stress prevention (art. 37, TU 81/208) and the evaluation of work-related stress are legal requirements for Italian companies, which would otherwise incur in breach of paragraph 1, article 29 of Law no. 81/2008.

Nomophobia is the uncontrolled fear of remaining disconnected from the mobile phone network. The symptoms range from simple anxiety (e.g., low battery or credit, lack of coverage, mobile forgotten) to panic attacks: shortness of breath, dizziness, tremors, sweating, fast heartbeat, chest pain and nausea.

## Conclusions

The technology dependency of labor today is still an underestimated phenomenon and not fully recognised within the psychological discomforts. Thus, it is diagnosed only when associated with other mental or physical problems, a state of affairs that currently is often diagnosed at an advanced stage, perhaps after a heart attack or other serious diseases, for which complete rest from working is prescribed.

Technostress, bullying, burn-out, and work-addiction are not diseases but psychosocial risk factors, that can potentially lead to the development of pathologies. Headache is the first cause of absence from work and one of the most common symptoms of the psychological distress related to working. Therefore, headache should not be underestimated because it can represent a predictive “red flag” of more complicated conditions. The mutual influence between headache and work activity is of growing interest in scientific research.

